# On the Cause and Treatment of Panniform Corneæ, Occurring with Granular Ophthalmia

**Published:** 1867-10

**Authors:** Jos. S. Hildreth

**Affiliations:** Chicago


### CHICAGO MEDICAL EXAMINER.

N. S. DAVIS, M.D., Editor.

VOL. VIII. OCTOBER, 1867. NO. 10.

### Original Contributions.

ARTICLE XLII.

### ON THE CAUSE AND TREATMENT OF PANNIFORM CORNEÆ, OCCURRING WITH GRANULAR OPHTHALMIA.

By JOS. S. HILDRETH, M.D., Chicago.

Read to the Illinois State Medical Society, June, 1867.

No disease of the eye is so universally prevalent as granular ophthalmia. None demands more skilful management. It is the parent of disorders producing the saddest results. Preëminent stand those which attack the cornea and darken the windows of life. To analyze their pathology and improve their treatment, should enlist our most earnest endeavors. The one selected for the subject of this paper is of absorbing importance.

A vascular and more or less opaque condition of the cornea is, technically, called "*pannus.*"

All keratic disease, accompanied by development of sanguiniferous vessels in the cornea, is termed panniform.

From being limited to a slight epanchement, with a few delicate vessels spanning the periphery, this affection may become a dense vascular exudation, firmly organized and involving the whole cornea. The primary cause may be:

1st. Mechanical, as with deviated cilia; when their removal from contact with the globe will abate corneal disturbance, if uncomplicated.

2d. Inflammatory action, producing solution of continuity, as phlyctenulæ, or abscess. As soon as restoration of the parts is completed, vascularity disappears from the cornea, in absence of other difficulty.

3d. Keratitis, consequent to corneal anæsthesia, without ntra-ocular pressure. Here the cornea will remain panniform, until the cause of its anæsthesia, and, thereby defective nutrition, has been removed.

4th. Mechanical and pathological disturbances may coöperate, as in granular ophthalmia.

The following cases represent diverse aspects of the affection in question. Their lids were granulated as nearly alike as possible, and general condition practically equal. With one, the corneæ were quite free from panniform encroachment, while with the other, they had become the seat of dense vascular exudation. That granular lids alone are not always sufficient to induce panniform disturbance in the cornea is, therefore, evident.

With the first patient, the pupils were fully susceptible to atropia, and its effects remained the ordinary length of time. The corneæ were also sensitive to the lightest touch. The cornea is to be tested by touching it lightly with the point of a camel's hair brush, previously wet and stripped quite dry, or a small twisted roll of unsized paper, also slightly moistened.

These symptoms are of peculiar import, as showing the corneæ had escaped becoming panniform, because they were endowed with sufficient vitality successfully to resist the constant friction of granulations upon them. Had the roughness of the thickened palpebral lining been firm enough to have disturbed the integrity of the corneæ, or, had their nutrition, from any cause, been defective, vascular development would have resulted.

With the second patient, the pupils, though somewhat obscured by the panniform exudation, were seen sufficiently well to mark their condition. Atropia could only partially take effect,* and its influence was of short duration. The corneæ, correspondingly, had lost their sensitiveness to touch. This last symptom, though influenced by the change of structure in the parts, is, however, of practical importance.

It is evident, panniform affections are consequent on defective nutrition of the membrane specially involved. The vascular development vicariously contributing to the temporary maintainance and the repair. When disposing causes are long continued, this vascular exudation, from being equal to the support and restoration of the impaired cornea, becomes so firmly organized as to constitute in and of itself an affection of difficult management.

As already stated, these causes may be mechanical and pathological. The first is due to friction of the granulated lids; the second, a general or local influence, or both combined; the one being a cachexia, the other a certain condition of the ciliary ring which, "without intra-ocular tension," lessens dilatability of the pupil, and so depresses the nervous integrity of the cornea as to produce anæsthesia and materially impair its nutrition. It thus becomes an agent to induction of panniform disease.

Though the general health be restored and granulations removed from the lids, the corneæ will make unsatisfactory improvement while this depressing influenee continues.

This affection of the ciliary ring, I have frequently found in kerastic diseases, and invariably present, to greater or less degree, in vascular exudation of the cornea, occurring with granular ophthalmia. When acute or spasmodic, it often persists until corneal disturbance is developed; relaxes again, under some exciting cause, to be renewed; then decline, and may thus continue until change of structure in both cornea and ciliary ring becomes so great as to demand surgical relief.

As restricted dilatability of the pupil and lessened effects of atropia are pathognomonic symptoms, so they become therapeu-

* Four grains of neutral sulphate of atropia to an ounce of distilled water, is the strength ordinarily employed. In some cases, a ten grain solution may be used.

tic guides. Corneal anæsthesia is also a concurrent sign; but, as before suggested, allowance must be made for exudations and other changes in structure, which tend to diminish tactile sensibility.

One of the most important steps in treatment for some forms of keratitis, especially panniform with granular lids, is to obviate the depressing influence upon the nutrition of the cornea, caused by that peculiar condition of the ciliary ring whose diagnosis has just been described. To obtain this result, the pupil must be kept well dilated until its full susceptibility to atropia returns and contraction is not followed by corneal anæsthesia. That state of the eye called atropinism must be avoided, by not using more atropia than is required to maintain dilatation. I have not observed it in this class of cases, but should it occur, some other mydriatic, such as hyoscyamus, may be employed for a time.

When, instead of mechanical complication on the part of the iris, persistent and energetic use of atropia produces but limited dilatation of the pupil and impartial relief of existing corneal anæsthesia, division of the ciliary ring becomes requisite. The operation should be performed so as thoroughly to divide what is comprised under the term of "ciliary muscle" or ring, from the insertion of the iris to its posterior limit. Atropia will then take effect, and, to secure full benefits of the operation, its use must be continued in the manner above described.

When synechia opposes dilatation, recourse may be had to the anæsthetized state of the cornea, in determining the effects of the drug, or of an operation. But mechanical complications should be obviated by appropriate means when required.

The local excitement following this operation is usually quite light. In cases of long standing, it may become considerable; though, in my own experience, not only free from danger, but productive of good results, in hastening resolution of the panniform exudation. As soon as the state of the eye will permit, stimulating applications within the lids should be employed. These are various: but from the bromide of ammonium, with tannin, and a mercurial ointment, I have obtained the best results.

℞. Bromide of Ammonium,------------ ʒss. to ʒj.

Tannin,---------------------------------------- ʒj.

Glycerine, (pure,)--------------------------- ℥j.

M.S.A.

To be applied by a camel's hair brush to the conjunctiva, the lids being everted.

℞. Red Precipitate,-------------------------- gr. vj.
Calomel,----------------------------------- gr. viij.
Sulph. Zinc,--------------------------
Camphor,--------------------------- āā gr. j.
Lard, (fresh,)---------------------------- ℥j.

Mix well.

A piece about twice the size of a hemp seed within the lids.

When the panniform exudation is dense and firmly organized, the operation of syndectomy is indicated. This consists of excision of a band of the conjunctiva, about four millimetres in width, from immediately around the periphery of the cornea. It should be so thoroughly removed as fully to expose the sclerotic beneath. The effect, of course, is to cut off all immediate connection between the conjunctiva and network of abnormal vessels in the cornea. But the results will be found more satisfactory if the ciliary ring, at the same time, be divided.

RESUME OF TREATMENT.

*a.* Full and maintained dilatation of the pupil until the close.

*b.* Stimulating applications within the lids every second day, or as often as the state of the parts will permit.

*c.* Division of the ciliary ring, to allow dilatation of the pupil, when it cannot otherwise be obtained.

*d.* Syndectomy in case the panniform exudation is too firmly organized for removal by medical means.

*e.* Appropriate constitutional measures.

Manifold experiment has demonstrated the efficacy of the above, for the relief of panniform disease of the cornea. Further, it has been noted that resolution of the granulations has been promoted by the means required to maintain proper dilatation of the pupil in such cases.

Favorable results are obtained by purulent inoculation of the conjunctiva. But the loathsome nature of the disease thus engendered, the danger of infection to attendants, and the uncertainty of results, render other treatment very desirable.

*Chicago, June 1st, 1867.*

